# Bioresponsive and near infrared photon co-enhanced cancer theranostic based on upconversion nanocapsules†
†Electronic supplementary information (ESI) available: Reactions between disodium maleate and water; photographs of POM; XPS and EDS spectra of the samples; Zeta potential testing results; TEM images of some samples; XRD spectra, FT-IR spectra and N_2_ absorption–desorption isotherm of the samples; standard curve and UV-vis spectra of DOX; degradation and drug release measurements of some samples; *in vitro* CT and MRI measurements of UCNPs@mSiO_2_; *in vitro* and *in vivo* assays of UCNPs@ySiO_2_–DOX; viabilities of L929 cells; and H&E stained images, blood biochemistry and hematology data of mice. See DOI: 10.1039/c7sc05414a


**DOI:** 10.1039/c7sc05414a

**Published:** 2018-02-06

**Authors:** Jiating Xu, Wei Han, Ziyong Cheng, Piaoping Yang, Huiting Bi, Dan Yang, Na Niu, Fei He, Shili Gai, Jun Lin

**Affiliations:** a Key Laboratory of Superlight Materials and Surface Technology , Ministry of Education , College of Materials Science and Chemical Engineering , Harbin Engineering University , Harbin , 150001 , P. R. China . Email: yangpiaoping@hrbeu.edu.cn; b State Key Laboratory of Rare Earth Resource Utilization , Changchun Institute of Applied Chemistry , Chinese Academy of Sciences , Changchun 130021 , P. R. China . Email: jlin@ciac.ac.cn; c College of Sciences , Northeast Forestry University , Harbin 150050 , P. R. China

## Abstract

Developing nanotheranostics responsive to tumor microenvironments has attracted tremendous attention for on-demand cancer diagnosis and treatment. Herein, a facile Mn-doping strategy was adopted to transform mesoporous silica coated upconversion nanoparticles (UCNPs) to yolk-like upconversion nanostructures which possess a tumor-responsive biodegradation nature. The huge internal space of the innovated nanocarriers is suitable for doxorubicin (DOX) storage, besides, the Mn-doped shell is sensitive to the intratumoral acidity and reducibility, which enables shell biodegradation and further accelerates the breakage of Si–O–Si bonds within the silica framework. This tumor-responsive shell degradation is beneficial for realizing tumor-specific DOX release. Subsequently, polyoxometalate (POM) nanoclusters that can enhance photothermal conversion in response to the tumor reducibility and acidity were modified on the surface of the silica shell, thereby achieving NIR-enhanced shell degradation and also preventing premature DOX leakage. The as-produced thermal effect of the POM couples with the chemotherapy effect of the released DOX to perform a synergetic chemo-photothermal therapy. Additionally, the shell degradation brings size shrinkage to the nanocarriers, allowing faster nanoparticle diffusion and deeper tumor penetration, which is significant for improving theranostic outcomes. Also, the drastic decline of the red/green (R/G) ratio caused by the DOX release can be used to monitor the DOX release content through a fluorescence resonance energy transfer (FRET) method. The MRI effect caused by Mn release together with the MRI/CT/UCL imaging derived from Gd^3+^/Yb^3+^/Nd^3+^/Er^3+^ co-doped UCNPs under 808 nm laser excitation endow the nanosystem with multiple imaging capability, thus realizing imaging-guided cancer therapy.

## Abstract 2

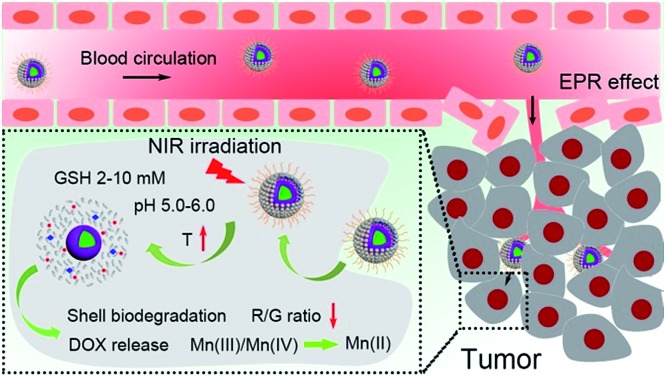
A yolk-like nanocapsule with responsiveness to tumor microenvironment and NIR photons was invented by integrating a tumor-responsive photothermal agent on Mn-doped UCNPs@mSiO_2_ nanospheres for multiple imaging guided thermo-chemotherapy.

## Introduction

On-demand cancer diagnosis and treatment triggered by intrinsic physiological microenvironments has received considerable attention in recent years. Such therapies can simultaneously reduce the damage of anticancer agents to normal tissues and improve their therapeutic efficacy.^1^–^5^ Among the various physiological parameters, reducibility and acidity are the characteristic statuses of the tumor tissues compared to normal tissues. It has been demonstrated that glutathione (GSH) is the most abundant reducing agent in the tumor, with a content at least 4-fold higher than that in normal tissues.^6^,^7^ In addition, the tumor pH of the extracellular microenvironment is about 7.2–6.5 depending on the tumor type and stage, while that of intracellular early endosomes and lysosomes reaches 6.2–5.0.^8^

Innovation of nanotheranostics posing effective therapy and diagnosis is highly desirable.^9^–^12^ To date, chemotherapy is the most widely used tool for cancer therapy.^13^–^18^ Recently, the integration of rare earth-based upconversion nanoparticles (UCNPs) with mesoporous silica shells for the delivery of chemotherapeutic agents to the tumor sites has attracted a great deal of attention due to the huge potency in realizing multimodal (*e.g.* X-ray computed tomography (CT), upconversion luminescence (UCL), magnetic resonance imaging (MRI), and so forth) imaging-guided chemotherapy.^19^–^23^ However, the limited drug delivery efficacy and dull drug release are two major obstacles in achieving satisfactory treatment outcomes. Besides, the traditional mesoporous silica coated UCNPs are size-unchanged at a tumor, thus they are usually restrained in the interstitium of a solid tumor after extravasation. Under these circumstances, the nanoparticles cannot reach as many cancer cells as possible, so accurate tumor delineation is hard to achieve. For ideal chemotherapeutic nanocarriers, high drug loading and minor side effects are significant in boosting the therapy outcome and avoiding drug resistance.^24^–^26^ Hence, a number of mesoporous silica related nanovehicles have been developed to achieve high storage and controlled release of chemotherapeutic agents.^27^ Nevertheless, the physicochemical stable –Si–O–Si– framework makes it hard to biodegrade in physiological conditions,^28^,^29^ thus causing incomplete drug release. The biodegradation of silica shells has not been efficiently achieved so far, which is the most critical hindrance in their practical application. Hence, it is still a formidable challenge to regulate the biodegradability of the shells on inorganic nanoparticles when used as chemotherapeutic drug carriers.

It is significant to give reliable tumor delineation before therapy.^30^–^32^ In recent decades, a growing amount of literature demonstrates that lanthanide-dope UCNPs hold great promise in combining multimodal bioimaging,^33^–^36^ which will greatly offset the defects of single-modality imaging.^37^,^38^ Upconversion is a multiphoton process, which transduces more than one low-energy excitation photon to a high-energy emission photon.^39^–^46^ This unique feature is especially beneficial for optical imaging because the long-wavelength photons have deeper penetration, allowing longer imaging distances. Besides, upconverted emission can be easily distinguished from the auto-fluorescence of biotissues,^47^ thus avoiding background interference during the visualization process. Furthermore, UCNPs doped with Gd^3+^ and Yb^3+^ (with unpaired electrons and high atomic numbers) ions are promising nanoprobes for MRI and CT imaging,^48^–^50^ which unite with the UCL imaging to offer whole-body visualization at high spatial resolutions and the real-time observation of nanocarriers in tumor tissues.^51^,^52^

It is known that PEGylation usually endows UCNP-based nanotheranostics with larger sizes, which makes them selectively accumulate in tumor lesions through the enhanced permeability and retention (EPR) effect.^53^,^54^ However, the large size is detrimental for nano-agents to penetrate into the tumor parenchyma deeply. After extravasation from the tumor vessels, the agents are mainly restricted to adjacent zones of the tumor vasculatures due to the dense extracellular matrix and high interstitial fluid pressure,^55^–^58^ therefore markedly limiting their penetration depth. These two contradictory aspects of inferior tumor penetration and improved tumor extravasation are intractable and uncompromising when using UCNP-based nanocarriers for anticancer theranostics. Actually, the dilemma also promotes the development of a size-variable nanosystem that could maintain a large size for longer blood circulation and selective tumor extravasation, while evolving into small-sized particles at the tumor for effective penetration and distribution.

Here, a facile Mn-doping method was used to endow a nanosystem with tumor-triggered shell biodegradation. It is known that Mn is an important element in human bodies for regulating metabolisms,^59^,^60^ and its uptake and excretion can be efficiently controlled by biological systems. In addition, the paramagnetic Mn(ii) ions can act as *T*_1_-weighted MRI contrast agents,^61^–^63^ which holds promise in uniting with the CT, MRI, and UCL imaging derived from UCNPs to perform tumor-improved multiple imaging. The –Mn–O– bonds are sensitive to reduction or acidity,^64^–^66^ hence the Mn-doped shell can degrade in the mild acidic and reductive tumor microenvironment due to the inherent breakup nature of the Mn–O bonds. The breakage of the Mn–O bonds and the subsequent release of Mn ions from the silica shell can produce a large amount of defects within the silica framework and further promote the shell degradation, including Si–O cleavage. Significantly, according to the law of Arrhenius, temperature is the only factor that affects the rate of a chemical reaction. So we envisage introducing a NIR-absorptive agent to enhance the internal temperature of the nanosystem, and further improve its biodegradation rate. In this research, for the first time, a polyoxometalate (POM) cluster with self-adaptive electronic structure for enhanced NIR absorption was modified on a silica shell to realize NIR-enhanced shell degradation. This tumor-responsive photothermal conversion of the POM cluster also endows the nanosystem with PTT function, which unites with the chemotherapy to perform a synergetic chemo-photothermal therapy. Furthermore, the disintegration of the Mn-doped shell can further improve the *T*_1_-weighted MRI thanks to the Mn release. Notably, the shell biodegradation brings an obvious size decrease to the UCNCs, thus making the exposed particles able to reach more cancer cells to achieve efficient tumor distribution. Due to the fluorescence resonance energy transfer (FRET) between the green emissions of the UCNPs and the DOX molecules, the green emissions are largely diminished. Therefore, the shell biodegradability-enhanced DOX release inevitably brings a rapid decline of the R/G ratio, which acts as a sensor for the FRET sensing of DOX release when used for *in vivo* cancer therapy.

## Results and discussion

**Scheme 1:**
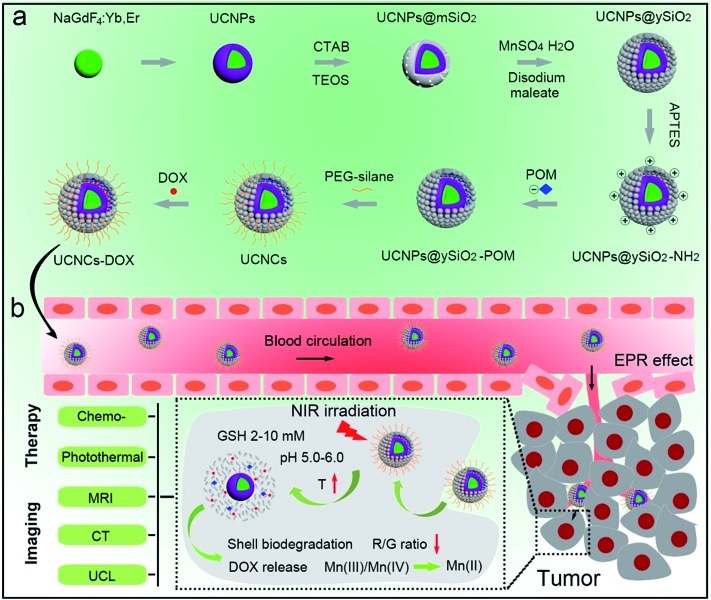
Schematic illustration for the synthesis of UCNCs–DOX (a) and the transport of UCNCs–DOX in a blood vessel, EPR-mediated tumor accumulation, tumor microenvironment and NIR photon co-enhanced cancer therapy, and multimodal imaging functions (b).

A schematic illustration for the synthesis of UCNCs–DOX is depicted in Scheme 1a. As shown, there are several important steps in the synthetic procedure. The coated active-shell (NaGdF_4_:Nd,Yb) is beneficial to achieve superior upconversion of the NaGdF_4_:Yb,Er core nanoparticles under 808 nm laser irradiation. After that, the UCNPs were coated with a shell layer of mesoporous silica (mSiO_2_), and then a gas–liquid template method was used to enable the formation of Mn-doped yolk-like upconversion nanostructures (UCNPs@ySiO_2_). After the POM nanoclusters were modified on the inner surface of the silica shell, the shell surface was modified *via* PEGylation and the resulting UCNCs were used to store DOX for multiple imaging-guided chemo-photothermal therapy. The addition of disodium maleate enables the formation of a mild alkalescent solution (Scheme S1,† reaction (1)). Under hydrothermal conditions, a small portion of silica from the inner shell was hydrolyzed to H_4_SiO_4_. Simultaneously, the active sites produced on the silica shell adsorbed carboxylate species and Mn^2+^ ions from the decomposition of disodium maleate. Subsequently, the carboxylate decomposed into gaseous species including CO_2_ under hydrothermal conditions (Scheme S1,† reaction (2)). Notably, the reaction between H_4_SiO_4_ and Mn^2+^ ions resulted in the deposition of Mn-doped silica on the liquid–gas interface, forming solid spheres on the shell. The same growth and deposition processes on the shell continued to produce unique yolk-structured nanocapsules with a nanospheres-stacked shell. Subsequently, a nano-sized photothermal agent of POM clusters was loaded on the inner surface of the silica shell through electrostatic interaction. Through these elaborate designs, a multimodal imaging-guided chemotherapeutic nanomedicine with tumor-responsiveness was obtained. As depicted in Scheme 1b, the nano-sized UCNCs–DOX was transported within blood vessels, and accumulated at tumor tissues *via* the EPR effect. The biodegradation of the silica shells can be easily triggered by mild acidic and reductive tumor microenvironments, causing the release of the Mn ions. The Mn release-triggered shell biodegradation can facilitate DOX release and improve the *T*_1_-weighted MRI imaging. Furthermore, the DOX release induces a decrease of the R/G ratio, which can be used for the sensing of the DOX release *via* a FRET method. Importantly, the tumor conditions can markedly enhance the 808 nm photon absorptivity of the POM to generate a large amount of heat, which can not only accelerate the above processes but also achieve a synergetic PTT modality.

**Fig. 1:**
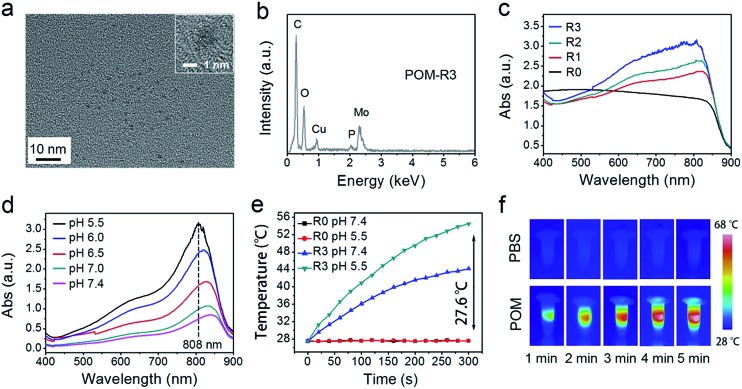
TEM image (the inset shows the corresponding high resolution image) (a) and the EDS spectrum (b) of the POM-R3 cluster. UV-vis absorption spectra of POM solutions at varied reduction states (c) and the POM-R3 solution at varied pHs (d). Photothermal heating curves of POM-R0 and POM-R3 solutions at pH 5.5 and pH 7.4 (e), and photothermal heating images (f) of POM-R3 (pH 5.5) and PBS buffer solutions under 808 nm laser irradiation (0.72 W cm^–2^).

In this study, a photothermal nanotransducer, consisting of the POM clusters, was used to improve the biodegradable behavior of the UCNCs and realize a combinational PTT under 808 nm laser irradiation, thus realizing NIR photon enhanced drug release and cancer therapy. In Fig. S1,† the color of the fabricated POM clusters changes from colorless to dark-blue with the reduction state changing from R0 to R3. Among them, the POM-R3 cluster is highly uniform with an average size of ∼1.8 nm at pH 7.4 as observed in the TEM image and the inset image in Fig. 1a. Their EDS spectrum in Fig. 1b, coupled with the XPS spectrum in Fig. S2,† demonstrates the existence of all of the essential chemical elements (Mo, P, and O) of the POM-R3 clusters. Subsequently, the UV-vis absorption property of the POM nanoclusters at various reduction states was tested to investigate the redox-activated absorption property. As depicted in Fig. 1c, the pure solution of POM-R0 exhibits low NIR absorptivity. As for the POM at the various reduction states, the absorption profiles show an obvious peak in the NIR range, which enhances gradually as the reduction state increases. The boosted reduction degree of POM-R0 will facilitate the occupied cation sites and delocalized electron density of Mo(v) through the reversible and multiple steps of electron exchange, which will simultaneously strengthen the electron relaxation polarization, resulting in enhanced NIR absorptivity.^67^ Also, the pH-responsive absorption of POM-R3 was observed, with the acidity enhancing the absorption ability of the POM-R3 clusters (Fig. 1d). The significant blue shift towards 808 nm couples with the reducibility and acidity co-enhanced NIR absorptivity, enabling the POM clusters to be a promising photothermal conversion agent under tumor conditions, which feature mild acidity and reducibility. After that, the photothermal conversion performance of the POM-R3 and POM-R0 solutions at pH 5.5 and 7.4 were investigated upon 808 nm laser irradiation. As displayed in Fig. 1e, the temperature of the acidic POM-R3 solution increased to 54.8 °C within 5 min of 808 nm laser irradiation, which is obviously higher than the value of 41.8 °C for POM-R3 under neutral conditions. As for the POM-R0 solution, it shows no temperature change for both the acidic and neutral solutions. The thermal images in Fig. 1f afford direct evidence for proving that the acidic POM-R3 is an efficient agent in converting 808 nm photons into thermal energy. The acidity and reducibility, which coincidentally are characteristic features of the tumor microenvironment, can co-enhance the photothermal conversion efficacy of the POM, implying their potency in bioresponsive photothermal fields. In other words, this nano-sized POM cluster holds promise in combining with chemotherapeutic nanoplatforms to achieve a tumor specific chemo-photothermal therapy.^68^

**Fig. 2:**
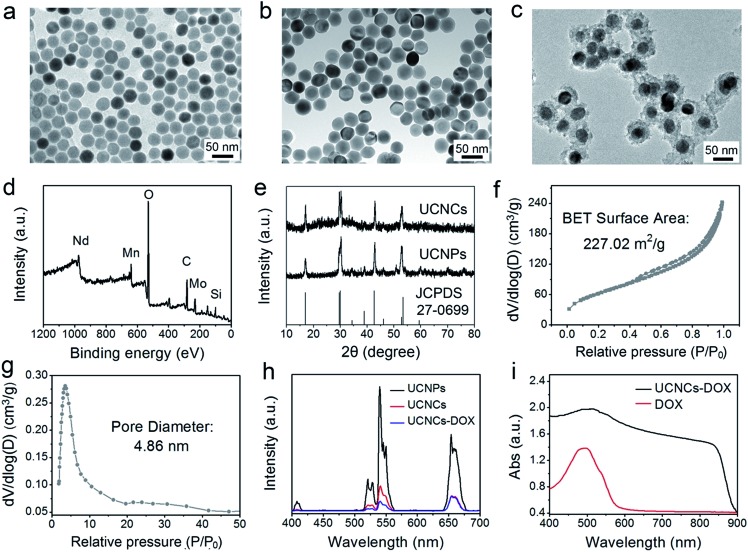
TEM images of NaGdF_4_:Yb,Er core nanoparticles (a), UCNPs (b) and UCNCs (c). An XPS spectrum of UCNCs (d). XRD patterns of the UCNPs and UCNCs (the standard patterns of β-NaGdF_4_ are also supplied) (e). The N_2_ absorption–desorption isotherm (f) and corresponding pore-size distribution of the UCNCs (g). Upconversion emission spectra of the UCNPs, UCNCs, and UCNCs–DOX (h). UV-vis absorption spectra of the DOX molecule and UCNCs–DOX (i).

Here, a biodegradable silica shell was coated on NaGdF_4_:Yb,Er@NaGdF_4_:Nd,Yb (labelled as UCNPs) for chemotherapy application. The core nanoparticles of NaGdF_4_:Yb,Er and the core–shell UCNPs were synthesized using a high-temperature decomposition method. After coating with mesoporous silica, the fabricated UCNPs@mSiO_2_ spheres were further treated under hydrothermal conditions, thereby obtaining yolk-like UCNPs@ySiO_2_. Before PEGylation, the POM clusters were inserted into the mesopores of the UCNPs@ySiO_2_ through the electrostatic interaction effect to obtain UCNCs. The Zeta potentials of the amino modified UCNPs@ySiO_2_ and POM-R3 were tested to be 13.6 mV and –22.5 mV, respectively (Fig. S3a and b†), so the positively-charged POM-R3 can be easily loaded into the surface of the silica shell. The Zeta potential of the UCNPs@ySiO_2_–POM decreased to 8.42 mV (Fig. S3c†). Fig. 2a–c respectively show the TEM images of the NaGdF_4_:Yb,Er core nanoparticles, UCNPs and the UCNCs. The TEM image in Fig. 2a reveals that the NaGdF_4_:Yb,Er core nanoparticles consist of uniform and monodispersed particles with a mean size of 32.8 nm. The TEM image in Fig. 2b implies that the dispersion and uniformity of the UCNPs have been well kept, and the mean size increased to 43.1 nm. After the mesoporous silica was coated on the UCNPs, the UCNPs@mSiO_2_ have a mean diameter of 60.7 nm (Fig. S4a†). Fig. S4b† displays the EDS spectrum of the UCNPs@mSiO_2_. As for the UCNPs@ySiO_2_ and the final obtained UCNCs, the particle size is about 65.8 nm (Fig. S5a† and 2c). The EDS spectrum exhibited in Fig. S4b† implies the successful Mn doping in silica. The XPS spectrum in Fig. 2d indicates the fine combination of UCNPs@ySiO_2_ and POM-R3. The XRD patterns of the UCNPs, UCNCs and the standard line of β-NaGdF_4_ (JCPDS no. 27-0699) are displayed in Fig. 2e.

Meanwhile, the XRD spectra of the as-prepared POM-R3 clusters, UCNPs@mSiO_2_, and UCNPs@ySiO_2_ are supplied in Fig. S6.† As shown, the diffraction peaks of β-NaGdF_4_ and the POM-R3 cluster are well maintained in the final UCNCs. The XRD pattern of the UCNPs@mSiO_2_ shows a broad diffraction peak of amorphous materials at 2*θ* = 22° beside the characteristic sharp peaks of the UCNPs, indicating successful silica coating. In addition, the XRD patterns of the UCNPs@ySiO_2_ and the later obtained sample exhibit a broadened peak of Mn_*x*_(SiO_4_)_*y*_ at about 2*θ* = 33°, indicating the covalent bonding of the Mn species within the silica framework.

FT-IR spectrophotometry was used to detect the functional groups on the sample in every step and supply additional evidence for the successful sample preparation. As depicted in Fig. S7,† the spectrum of the POM shows the bands between 1100–400 cm^–1^ originating from the asymmetric stretching vibrations of the P–O and Mo–O bonds. For oleic acid (OA)-stabilized UCNPs, the spectrum exhibits bands at 1463 cm^–1^ and 1564 cm^–1^ associated with the vibrations of the carboxylic groups, and the broad band at around 3450 cm^–1^ derives from the stretching vibration of O–H. The strong transmission bands at 2854 cm^–1^ and 2924 cm^–1^ are attributed to the asymmetric and symmetric stretching vibrations of –CH_2_. With the mesoporous silica shell coating, the FT-IR spectrum of the obtained UCNPs@mSiO_2_ has characteristic peaks at 3432 cm^–1^ and 947 cm^–1^, implying that the sample surface has a large number of OH groups, which is advantageous for adsorbing drug molecules through hydrogen bonds. Additionally, the peaks at 802 cm^–1^ and 1088 cm^–1^ are derived from the vibration of Si–O–Si bonds. The FT-IR spectrum of the UCNPs@ySiO_2_ is similar to that of the UCNPs@mSiO_2_ due to the similar functional groups. However, there are additional peaks at around 1500 cm^–1^, which are caused by the vibrations of Mn–O bonds. After PEGylation, a strong C–O stretching vibration is detected on the surface of the UCNCs. For UCNCs–DOX, the peaks between 1000–1800 cm^–1^ are caused by the DOX molecules. The N_2_ adsorption/desorption isotherms of the UCNPs@ySiO_2_ and UCNCs–DOX are displayed in Fig. S8a and b,† respectively. As shown, the UCNPs@ySiO_2_ possess typical type IV isotherms, implying the mesoporous structure of the silica channels. This yolk-structured sample possesses a relatively high Brunauer–Emmett–Teller (BET) surface area of 303.44 m^2^ g^–1^ with a high pore volume of 0.58 cm^3^ g^–1^. The large surface area is ideal to store the POM-R3 nanocluster PTT agent and DOX chemotherapy agent. Subsequently, after the UCNPs@ySiO_2_ were decorated with the POM, the BET surface area decreased to 227.02 m^2^ g^–1^ with a pore volume of 0.39 cm^3^ g^–1^, and a mean pore size of 4.86 nm (Fig. 2f and g). The silica channels of the final UCNCs still possess mesoporous structure, and the pore size is suitable for DOX loading. The BET surface area is reduced to 27.32 m^2^ g^–1^ with a pore volume of 0.06 cm^3^ g^–1^, which validates the DOX loading in the silica channels (Fig. S8b†). Fig. 2h exhibits the upconversion emission spectra of the UCNPs, UCNCs and the final obtained UCNCs–DOX. Under 808 nm laser excitation, there are three characteristic emission peaks of Er^3+^ at 630–680 nm (^4^F_9/2_ → ^4^I_15/2_), 530–570 nm (^4^S_3/2_ → ^4^I_15/2_), and 510–530 nm (^2^H_11/2_ → ^4^I_15/2_). In comparison with the UCNPs, the emission intensity of the UCNCs was decreased obviously, perhaps due to the quenching effect derived from the Mn-doped silica shell. For the UCNCs–DOX, it has a lower emission intensity than the UCNCs, especially in the green region, which is caused by the strong absorption by DOX in the green range (Fig. 2i). The UV-vis absorption profile of the final UCNCs–DOX also has an obvious absorption peak from 450 nm to 550 nm, which is originated from the loaded DOX molecules. According to the Lambert–Beer Law and the testing results displayed in Fig. S9,† the DOX loading rate of the UCNCs is calculated to be 54.3%. Actually, there is also an absorption peak (800 to 900 nm), which is caused by the modified POM cluster.

**Fig. 3:**
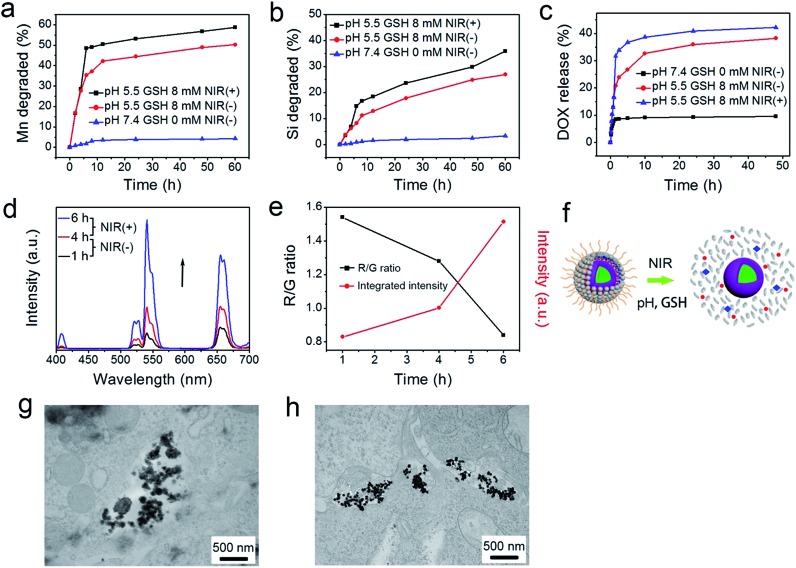
Accumulated release profiles of biodegraded Mn (a) and Si (b) in PBS (pH 7.4 and GSH 0 mM, and pH 5.5 and GSH 8 mM) without and with NIR laser irradiation from 4 to 8 h. DOX release profiles for UCNCs–DOX in PBS (pH 7.4 and GSH 0 mM, and pH 5.5 and GSH 8 mM) without and with NIR laser irradiation from 1 to 5 h (c). Upconversion emission profiles of UCNCs–DOX as a function of release time in PBS (pH 5.5, and GSH 8 mM) (d). The integrated emission intensity and the R/G ratio *versus* release time (e). Schematic illustration of DOX release from UCNCs–DOX along with the shell biodegradation of the silica shell (f). Bio-TEM images of HeLa cells incubated with UCNCs for 2 h without (g) and with (h) irradiation under 808 nm laser for 10 min.

**Fig. 4:**
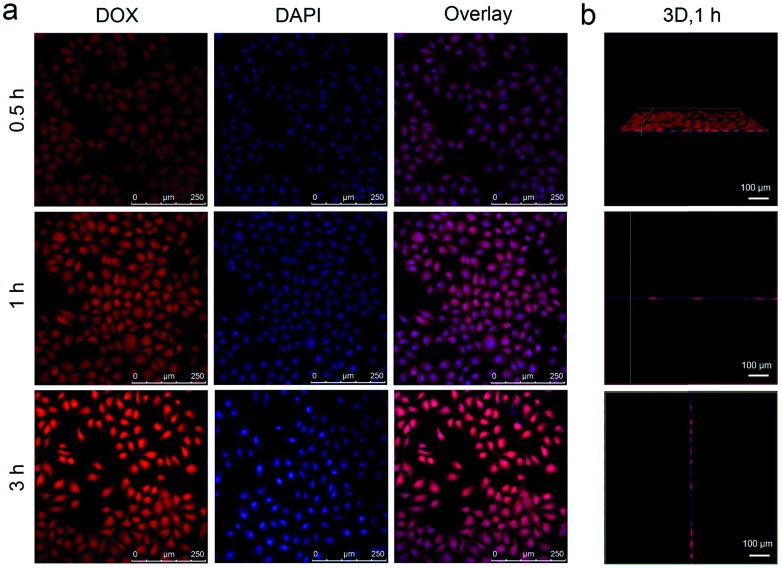
CLSM images of HeLa cells incubated with UCNCs–DOX for 0.5, 1, and 3 h at 37 °C (a), and a 3D fluorescence reconstruction of *Z*-axis-scanned images of HeLa cells incubated with UCNCs–DOX for 1 h (b).

As accepted, the Mn–O bonds are sensitive to the reductive and mildly acidic tumor microenvironment. Thus we envisage that the introduced Mn–O bonds within the silica framework of the silica can be disintegrated under tumor-like conditions, which can accelerate the biodegradation of the silica shell. In the tumor microenvironment, the extracellular pH is about 7.2–6.5, depending on the tumor stage and type, while that of intracellular early endosomes and lysosomes reaches 6.2–5.0. Besides, the intracellular microenvironment features a reductive nature with a GSH content at least 4-folds higher than that in normal tissues. To simulate the tumor microenvironment, a PBS buffer solution with pH 5.5 and GSH 8.0 mM was used for detailed biodegradation assays. PBS buffer with pH 7.4 and GSH 0 mM was used to simulate normal body conditions as a comparison. The UCNCs were dissolved in various PBS solutions, and the degradation processes were monitored using ICP tests. As depicted in Fig. 3a, when compared with that in neutral and none-reductive PBS, the release of Mn is obviously accelerated under mild acidic and reductive conditions. Moreover, the fast Mn release from the shells of the UCNCs accelerated the release of Si (Fig. 3b). Importantly, the 808 nm laser irradiation during the degradation time of 4 h to 8 h accelerated the Mn and Si release. So it can be concluded that the tumor acidity and reducibility can trigger the shell breakage of the Mn–O bonds and initialize shell degradation. Importantly, NIR photons have an accelerating effect on the shell degradation rate. The Si/Mn release profiles indicate that the breakup of the Mn–O bonds in acidic conditions induces the fast breakage of the –Si–O–Si– framework afterwards, and the NIR irradiation acts as an accelerator for enhancing shell collapse. Fig. S10a† demonstrates that the UCNPs@mSiO_2_ nanoparticles without Mn have no obvious degradation. For comparison, the degradation property of the UCNPs@ySiO_2_ has been investigated in different PBS solutions without and with NIR irradiation. As shown in Fig. S10b and c,† the NIR light irradiation nearly has no influence on the degradation rate of Mn and Si elements from the UCNPs@ySiO_2_, which validates the advantages of the POM cluster in promoting shell degradation. The TEM image in Fig. S11† validates that the shells of the UCNCs nearly disappear after the NIR irradiated degradation in reductive and acidic PBS solution. Encouraged by the biodegradable behavior of the UCNCs, the drug release profiles of the UCNCs–DOX were tested. As shown in Fig. 3c, the mild acidic and reductive PBS enables quicker drug release. Besides, the DOX release shows much enhanced efficacy in concurrent acidic and reductive conditions when irradiated using a NIR laser from 1 to 5 h during the releasing process, which is caused by the faster shell biodegradation. Also, the DOX release property of the UCNPs@ySiO_2_–DOX was investigated in different PBS solutions without and with NIR irradiation (Fig. S12†). Obviously, the NIR irradiated UCNCs–DOX has a higher DOX release rate than the NIR irradiated UCNPs@ySiO_2_–DOX. In other words, the thermal effect generated by the POM is advantageous for promoting DOX release. Upconversion emission spectra of the UCNCs–DOX in different PBS buffer solutions without and with NIR irradiation *versus* release time are shown in Fig. 3d. Because of the FRET between the UCNPs and the DOX, the green-emissive peaks of the UCNPs are greatly inhibited. It is known that the FRET effect will disappear when the distance between the donor and the acceptor is more than 10 nm. So with the release of DOX enhanced, the emission intensity of the green peaks increased faster than that of the red emissions, and the enhancement of the integrated intensity is mainly caused by the reduced quenching effect from the shell. Notably, the emission recovery, especially in the green range, during 1 to 4 h without NIR irradiation is higher than that during 4 to 6 h with NIR irradiation, which also validates that the NIR laser can enhance the shell degradation and the subsequent DOX release. The efficient biodegradability of the silica shell brings a rapid enhancement of the integrated emission intensity. In particular, the biodegradation-enhanced DOX release leads to a faster recovery of the green-emissive peaks than the red-emissive peaks, further leading to a rapid decline of the R/G ratio, which can be used to reflect the DOX release. This NIR and tumor conditions co-enhanced DOX release is very beneficial for cancer theranostics: (1) the shell degradation-enhanced DOX release enables efficient DOX release; (2) the adopted method is much easier than the construction of nanovalves in the mesopores of shells for on-demand drug release; and (3) the 808 nm light simultaneously excites the UCNPs for bioimaging and the POM for PTT. Fig. 3e depicts the variations of the integrated emission intensity and the R/G ratio of the nanosystem *versus* release time. It can be easily concluded that the efficient shell degradability brings a rapid enhancement of the integrated emission intensity. Moreover, the biodegradation-enhanced DOX release results in a faster recovery of the green-emissive peaks than the red-emissive peaks, thus leading to a rapid decline of the R/G ratio (from 1.54 to 0.84 during a release time of 6 h). In particular, the NIR laser irradiation plays a positive role in accelerating the DOX release due to the increased rate of the emission intensity and the R/G ratio during 4 to 6 h being more obvious than that during 1 to 3 h. The UCL status of the UCNCs–DOX can be used to reflect the drug release when used for *in vivo* cancer therapy. This nanomedicine, with tumor conditions and NIR photon co-enhanced biodegradation, is very advantageous for cancer therapy (Fig. 3f). On one hand, the tumor acidity and reducibility markedly enhances the NIR absorptivity of the POM, which enables a tumor-enhanced PTT modality. On the other hand, the tumor microenvironment and NIR photons co-enhance the shell degradation, which further enables the fast DOX release and thereby realizes highly-efficient chemotherapy. Moreover, the shell degradation decreases the dynamic diameter of the nanosystem, which enables efficient distribution and penetration in the tumor tissues, thus achieving superior bioimaging results. To study the intracellular biodegradation of the UCNCs, HeLa cancerous cells were incubated with UCNCs without and with NIR laser irradiation for 10 min during 2 h incubation. As displayed in Fig. 3g and h, the UCNCs can be easily endocytosized into the cell cytoplasm. Notably, the shell portion remained during the incubation time of 2 h without NIR irradiation (Fig. 3g), while the degradation of the shell can be clearly found in the bio-TEM image after the 2 h intracellular uptake with NIR irradiation (Fig. 3h), validating the intracellular biodegradation of the silica shells of the UCNCs. The cell uptake behavior of the developed sample was also investigated by incubating HeLa cells with UCNCs–DOX for 0.5, 1 and 3 h at 37 °C, with the corresponding CLSM images exhibited in Fig. 4a. DAPI, which can radiate blue emission, was used to mark the cell nuclei, and the DOX loaded in the nanosystem emits red fluorescence when excited using a 488 nm laser. Correspondingly, the merged images of the above two channels are provided. As shown in the first 0.5 h, there is only feeble red emission, which indicates that only a slight amount of the particles have been swallowed by the cells. When the incubation time is prolonged, the red signal becomes stronger, implying more particles are localized in the cells. These results demonstrate that the as-obtained nanomedicine can be efficiently internalized by HeLa cells. The intracellular accumulation of the UCNCs–DOX is further demonstrated using a three dimensional (3D) fluorescence reconstruction of *Z*-axis-scanned CLSM images where the nanomedicine could be clearly seen in the cytoplasms (Fig. 4b).

**Fig. 5:**
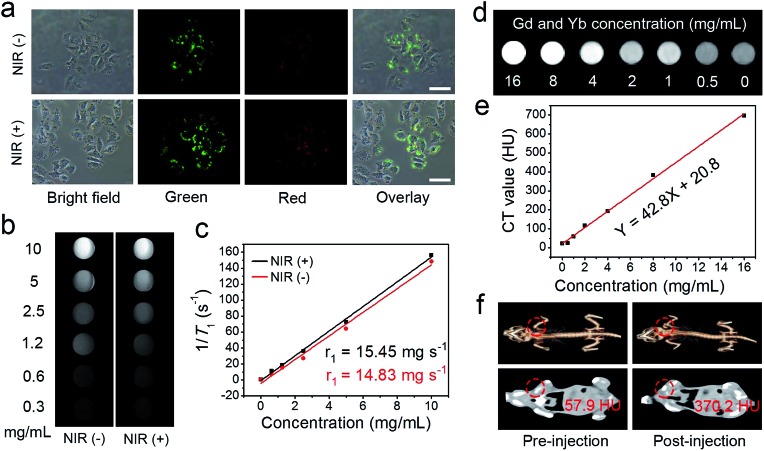
UCL microscopy images of HeLa cells incubated with UCNCs–DOX at 37 °C for 1 h without and with NIR irradiation. Scale bar: 50 μm (a). *In vitro T*_1_-weighted MR images of UCNCs incubated with PBS (pH 5.5, and GSH 8 mM) for 24 h without and with NIR laser irradiation (b) and corresponding relaxation rate *r*_1_*versus* sample concentrations (c). *In vitro* CT images of UCNCs at varied Gd/Yb concentrations (d) and corresponding CT values *versus* Gd/Yb concentrations (e). *In vivo* CT images of tumor-bearing mice before and after UCNCs injection (f).

Recently, the combination of multimodal imaging in a nanosystem has attracted tremendous attention for the construction of cooperative diagnostic agents. Here, the UCL imaging property of the UCNCs–DOX has been investigated intracellularly. As shown in Fig. 5a, two groups of HeLa cells were incubated with UCNCs–DOX at 37 °C for 1 h without and with 808 nm laser irradiation (0.72 W cm^–2^, for 5 min) during the incubation process. Obviously, the nanoparticles in the cell radiate luminous upconverted emissions (green and red) upon 808 nm laser excitation and the emissions are brighter for the NIR light irradiated group. In particular, the intensity enhancement of the green emission is more evident. The self-adaptive electronic structure of the POM endows the UCNCs–DOX with enhanced NIR absorptivity and further improves its photothermal conversion efficacy, thus generating internal heat in the nanosystem. The heat will inevitably increase the diffusion rate of the DOX in the silica channels, and importantly, promote the shell biodegradation. Due to the shell degradation and the DOX release, the quenching effect caused by the silica shell and the FRET effect between the DOX and green emission diminished, so the phenomenon in Fig. 5a is observed. The shell degradation has the following contributions: (1) liberation of the DOX release is considerably helpful for improving the chemotherapy outcome; (2) the decrease of the particle size makes the nanoparticles able to penetrate the tumor tissues deeply; (3) recovery of the UCL emission and the release of Mn^2+^ are respectively beneficial for achieving superior UCL and MRI imaging; and (4) the generation of the local hyperthermia acts as a synergetic PTT modality. The green emission obviously becomes stronger, while the red emission only has slight enhancement, which further validates that the nanomedicine can be utilized for R/G-based FRET sensing of drug release. Besides, there is no fluorescence signal detected outside of the cells, whereas the signal located at the intracellular region implies that the as-prepared sample has been internalized into the cells instead of merely being stained on the surface of the membranes. Moreover, the UCL signal is mainly located at the cell cytoplasm, which validates that the nanoparticles are engulfed by endosomes and lysosomes during endocytosis into the cells rather than passive adsorption. These results imply that the invented nanostructure with UCNPs loaded inside is an effective contrast agent for *in vitro* UCL imaging with ignorable background.

According to reports, the paramagnetic ions of Mn^2+^ and Gd^3+^ have a positive enhancing effect on *T*_1_ MRI signals and the most stable valence state of element Mn is Mn^2+^, so we envisage that the integration of the NaGdF_4_-based UCNPs and the Mn-doped silica shell poses superior *T*_1_ MRI imaging performance. Firstly, the *in vitro* MRI nature of the UCNPs@mSiO_2_ was measured (Fig. S13a†), and the longitudinal relaxivity *r*_1_ was calculated to be 13.85 mg^–1^ s^–1^ (Fig. S13b†). Subsequently, the *T*_1_-weighted MRI property of the UCNCs after being incubated for 24 h in PBS (pH 5.5, and GSH 8.0 mM) without and with NIR irradiation was studied. Fig. 5b displays the *in vitro* MRI images of these two groups. In Fig. 5c, due to the degraded paramagnetic Mn centers, the initial longitudinal relaxivity *r*_1_ of the UCNCs in acidic and reductive PBS without NIR irradiation is as low as 14.83 mg^–1^ s^–1^ but increased markedly to 15.45 mg^–1^ s^–1^ in reductive and acidic PBS under NIR irradiation. These results also demonstrate that the Mn-doping in the UCNPs@mSiO_2_ results in a slight increase of the MRI signal (from 13.85 to 14.83 mg^–1^ s^–1^). Significantly, the NIR-improved MRI effect is caused by the shell biodegradation of the UCNCs, which enhances the interaction probability of the Mn(ii) paramagnetic centers with water molecules. Besides, the MRI effect is derived from the exposed UCNPs together with that of Mn(ii) performing improved MRI. Therefore, UCNCs can act as tumor-responsive MRI contrast agents. As is known, the CT imaging technique is reliable because it affords deep tissue penetration and high-resolution three dimension structure details. Lanthanide-doped nanomaterials have been investigated extensively as CT imaging contrast agents due to the high atomic number of the rare-earth elements. Herein, the *in vitro* and *in vivo* CT imaging performances of the UCNCs are studied. As presented in Fig. 5d, the intensity of the CT signal increases markedly with the increase in Gd/Yb concentration. Besides, the CT values show positive enhancement *versus* the sample concentration, with a slope of 42.8 (Fig. 5e). The *in vivo* CT imaging was conducted on tumor-bearing mice without and with the injection of UCNCs. As shown in Fig. 5f, the post-injection tumor site has a CT value of 370.2 Hounsfield Unit (HU), which is obviously higher than that of the pre-injection tumor (57.9 HU). In Fig. S13c and d,† the *in vitro* CT imaging tests of the UCNPs@mSiO_2_ imply that Mn doping presents no obvious influence on the CT imaging signal of the UCNCs.

**Fig. 6:**
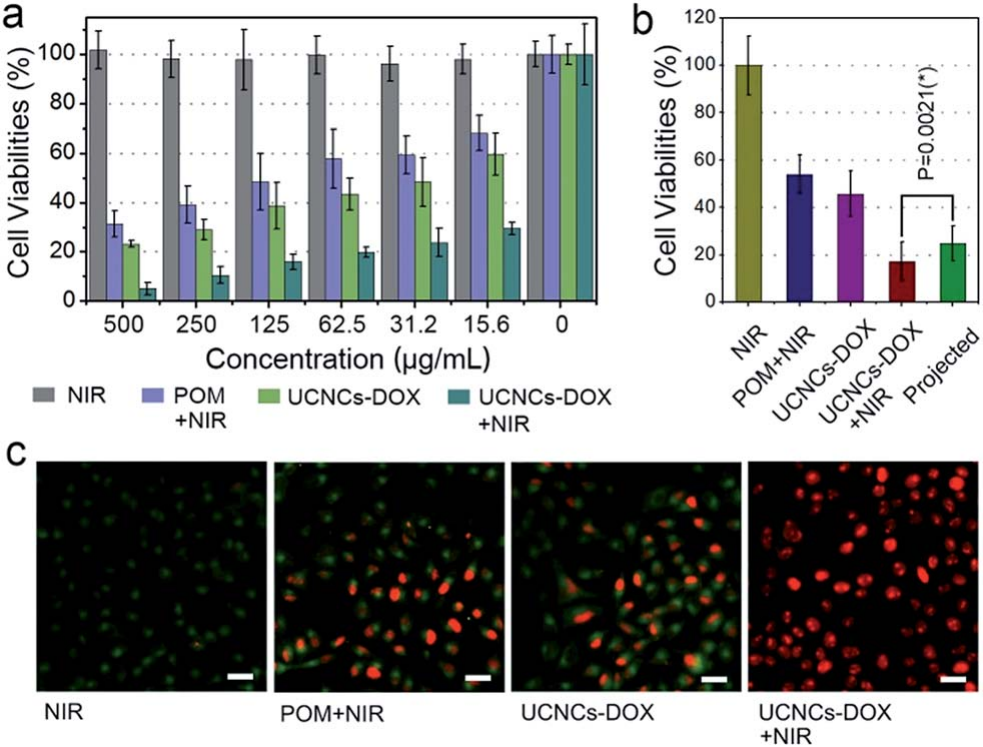
*In vitro* HeLa cell viabilities after various treatments (a), with the synergistic therapeutic effect of HeLa cells that have taken up UCNCs–DOX projected to various treatments. Statistical analysis was performed using the Student’s two-tailed *t* test (*P < 0.05) (b), and CLSM images of HeLa cells after various treatments, dyed with AM and PI. Scale bar: 50 μm (c).

Before practical application, the biocompatibility of the POM and the UCNCs to L929 fibroblast cells was evaluated using a standard MTT method, and the results are displayed in Fig. S14.† As shown, both of these two samples show high cell viabilities (>85%) in the whole concentration range even at 500 μg mL^–1^ after 24 h incubation, indicating that the samples have low-toxicity. In addition, to compare the anticancer efficacy of the prepared samples, four groups of HeLa cells were treated under different conditions for 24 h and then the cell viabilities were quantitatively tested using the MTT method (Fig. 6a). The viability of the cells treated with NIR laser only indicates that the 808 nm light shows no negative impact on HeLa cells. After POM incubation and then 808 nm light irradiation, a number of HeLa cells are killed with an obviously lower viability than those treated with laser irradiation only, which is caused by the PTT effect derived from the POM cluster. Then, when the cells were treated with a UCNCs–DOX sample, the viabilities are lower than those in the POM and NIR laser treated group, which is due to the chemotherapy effect derived from the released DOX molecules. Obviously, the UCNCs–DOX and NIR light treated group has the lowest cell viabilities (∼5%), which verifies that the chemotherapy and PTT combined nanosystem makes good utilization of the 808 nm photons to realize a synergistic cancer therapy. An additional assay was conducted to study the cell killing efficiency of UCNPs@ySiO_2_–DOX without and with NIR irradiation. As shown in Fig. S15a,† the NIR laser has no influence on the cell killing efficiency of the UCNPs@ySiO_2_–DOX because of the lack of the POM, and the corresponding killing rate is much lower than that of the NIR irradiated UCNCs–DOX. A Student’s two-tailed *t* test was performed to validate the synergistic effect of the NIR-triggered PTT and NIR-improved chemotherapy. As shown in Fig. 6b, four groups of cancer cells were subjected to different treatments. The projected value is achieved by multiplying the cell viability of the POM + NIR group by that of the UCNCs–DOX group. It was found that the group treated with UCNCs–DOX incubation and NIR irradiation has the lowest cell viability, even lower than the projected additive value (24.8%, and *P* = 0.0213), indicating the synergistic therapy effect. In Fig. 6c, PI, which can mark dead cells with a red color, and calcein AM, which can mark living cells with a green color, were used to dye HeLa cells under various treatments to prove the cell killing effect. As shown, in the CLSM image of HeLa cells in culture irradiated with an 808 nm laser, nearly all of the cells are green, indicating that NIR irradiation has no detrimental effect on the cells. For HeLa cells treated with POM incubation and NIR irradiation, a portion of red cells are observed, suggesting the PTT effect of the POM cluster under NIR irradiation. When incubated with UCNCs–DOX, some red cells can be seen due to the tumor-triggered DOX release. For cells incubated with UCNCs–DOX and irradiated with NIR light, nearly all of the cells are dyed red by PI, meaning that very few cancer cells survived due to the synergetic chemo-photothermal therapy. These results are in good agreement with Fig. 6a.

**Fig. 7:**
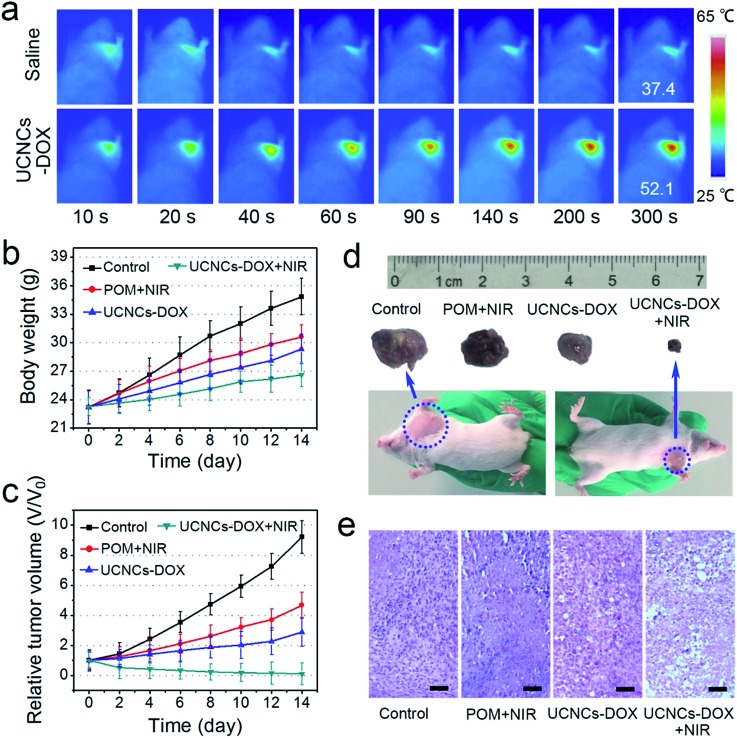
Photothermal images of U14 tumor-bearing mice before and after the injection of UCNCs–DOX upon 808 nm laser irradiation for various durations (a). Changes in the body weights (b) and relative tumor volumes (c) achieved for the mice under various treatments. Photographs of mice and excised tumors (d), and H&E stained images of tumor tissues obtained after 14 days of treatment. Scale bar: 100 μm (e).

Mouse experiments were conducted to investigate the antitumor efficiency of the UCNCs–DOX *in vivo*. Here, the first group of U14 tumor-bearing mice were treated with a saline injection as a control group, the second group was treated with a POM injection and NIR irradiation, the third group was injected with UCNCs–DOX, and the fourth group was injected with UCNCs–DOX and irradiated with NIR light. The mean body weights and relative tumor volumes of the mice were recorded every two days after the initial therapy. All formulations were intravenously administered through the tail vein. Fig. 7 presents the corresponding results of the *in vivo* antitumor experiments. *In vivo* photothermal imaging was performed under the exposure of an 808 nm laser for 5 min at 4 h post-injection of the UCNCs–DOX. The tumor temperature and thermal images were visualized with a thermal camera. As shown in Fig. 7a, the temperature of the tumor rapidly increased to 52.1 °C in 5 min under NIR laser irradiation (0.72 W cm^–2^), which was sufficient to thermally ablate the tumor, while the control group showed only a limited temperature increase. In Fig. 7b, the body weights of these four groups are not evidently affected over the investigation period, demonstrating that the samples nearly have no adverse effects on the mice. Compared with the control group, the POM + NIR group has the lower tumor growth speed, which demonstrates the PTT effect of the POM upon NIR irradiation. The tumor growth of the UCNCs–DOX injected group is obviously inhibited over the course of 14 days of treatment, which is due to the tumor microenvironment-induced DOX release, and the further chemotherapy effect to the cancer cells after extravasation at the tumor sites through the EPR effect. It is found that the NIR irradiated UCNCs–DOX shows the highest tumor inhibition efficacy compared to the other groups, which is derived from the NIR photon and tumor conditions co-enhanced chemo-photothermal therapy effect of the UCNCs–DOX. The NIR photons can be converted by the POM clusters (adapted to be NIR-absorptive in the tumor microenvironment) to obvious heat, which not only promotes the shell biodegradation and thereby realizing efficient DOX release, but also act as a synergetic PTT modality. The tumor in the fourth group is shrunken dramatically and its size is even smaller than the initial size, confirming that the NIR irradiation plays a key role in the aspect of boosting antitumor activity (Fig. 7c). Fig. S15b† implies that the tumor volumes of the UCNPs@ySiO_2_–DOX injected mice without and with NIR irradiation are almost the same, and they are bigger than those of the UCNCs–DOX + NIR treated group after 6 days of therapy, confirming the advantages of the POM decorated UCNCs–DOX in achieving a synergetic thermo-chemotherapy. Photographs (Fig. 7d) of representative mice and excised tumors also confirm that the tumor size upon UCNCs–DOX injection and NIR irradiation is the smallest, indicating the highest antitumor efficacy among the investigated groups. As a further proof assay, hematoxylin and eosin (H&E) stained tumor sections exhibited in Fig. 7e show the highest tumor damage degree for the mice treated with UCNCs–DOX injection and NIR irradiation, revealing good consistency with the above tumor growth data.

Pathomorphological analysis of the main organs including the spleen, kidney, heart, lung and liver is provided in Fig. S16.† There is no evident damage to the checked organs in the four groups, indicating a high *in vivo* biocompatibility for the UCNCs–DOX. Furthermore, the biochemical results for the UCNCs including total protein (TP), creatinine (CRE), alanine transaminase (ALT), blood urea nitrogen (BUN), and aspartate transaminase (AST), which is closely related to the liver and kidney functions, are exhibited in Table S1.† In comparison with the control group, there is no obvious injury of the kidney or liver. In addition, the complete blood tests show no distinct interference with the physiological regulation of haem or the immune response. All in all, the obtained nanocarrier presents huge potency for anticancer application.

## Conclusions

In summary, a novel yolk-like nanocapsule with bioresponsiveness was developed for NIR photon and tumor microenvironment co-enhanced theranostics. The developed UCNCs with UCNPs and POM clusters loaded inside the biodegradable shells have a large inner space, which is significantly ideal for DOX loading, and the unique structure was simply constructed by treating UCNPs@mSiO_2_ under hydrothermal conditions. The adopted Mn-doping method enables the biodegradation of the silica shells in mildly reductive and acidic tumor conditions by breaking the Mn–O bonds and subsequently initializing Mn release, which further induces the degradation of the shells. Then, the nano-sized POM clusters were modified on the silica shells to act as tumor-responsive photothermal transducers to simultaneously realize NIR-enhanced shell degradation and a synergetic PTT. Significantly, the shell biodegradation not only promotes DOX release but also enhances *T*_1_-weighted MRI due to releasing Mn, thus realizing tumor microenvironment and NIR photon co-improved cancer theranostics. The DOX release brings a rapid decline to the R/G ratio, which acts as a sensor for the FRET sensing of drug release. Under NIR laser irradiation, the UCNCs–DOX possesses tri-modal (CT, MRI, and UCL) imaging capabilities and high anticancer efficacy, revealing their potency in imaging-guided cancer therapy.

## Experimental section

### Reagents and materials

Hexaammonium molybdate tetrahydrate ((NH_4_)_6_Mo_7_O_24_·4H_2_O), sodium dihydrogen phosphate dodecahydrate (NaH_2_PO_4_·2H_2_O), hydrochloric acid (HCl), Er_2_O_3_ (99.99%), Nd_2_O_3_ (99.99%), Gd_2_O_3_ (99.99%), Yb_2_O_3_ (99.99%) and sodium fluoride (NaF) were acquired from the Sinopharm Chemical Reagent Co., Ltd. Oleic acid (OA), 3-4,5-dimethylthiazol-2-yl-2,5-diphenyl tetrazolium bromide (MTT), 1-octadecene (ODE), 4′,6-diamidino-2-phenylindole (DAPI), l-ascorbic acid, disodium maleate, doxorubicin (DOX), propidium iodide (PI), calcein AM, and methoxy PEG silane (*M*_w_ = 2000) were acquired from Sigma-Aldrich. Tetraethyl orthosilicate (TEOS), cetyltrimethyl ammonium bromide (CTAB), 3-aminopropyltriethoxysilane (APTES) and ammonium nitrate (NH_4_NO_3_) were acquired from the Tianjin Kermel Chemical Reagent Co., Ltd. Manganese(ii) sulfate monohydrate (MnSO_4_·H_2_O), trifluoroacetic acid (CF_3_COOH), and sodium trifluoroacetate (CF_3_COONa) were acquired from the Beijing Chemical Reagent Co. All of the chemical reagents used in this work were of analytical grade without further purification.

### Synthesis of NaGdF_4_:Yb,Er@NaGdF_4_:Nd,Yb (noted as UCNPs)

Briefly, 1 mmol of lanthanide oleates (Er/Yb/Gd = 2 : 18 : 80) and 5 mmol of NaF were mixed in a three-necked bottle with 15 mL of OA/ODE (v/v = 1 : 1). Then, the mixture was heated to 110 °C and degassed for 0.5 h. After being flushed with N_2_, the system was heated to 300 °C and maintained for 1 h. When the solution was cooled down to about 40 °C, ethanol and cyclohexane were used to centrifuge the solution, and the product was dispersed in cyclohexane. Subsequently, 0.05 mmol of Yb(CF_3_COO)_3_, 0.15 mmol of Nd(CF_3_COO)_3_, 0.3 mmol of Gd(CF_3_COO)_3_ and 1 mmol of CF_3_COONa were mixed with core nanoparticles, then 15 mL of OA/ODE (v/v = 1 : 1) was added. The mixture was heated to 120 °C and degassed for 1 h. After being flushed with N_2_, the system was heated to 310 °C and maintained for 1 h. The obtained sample was dispersed in cyclohexane (10 mg mL^–1^).

### Synthesis of UCNPs@mSiO_2_

Typically, a beaker with CTAB (0.1 g) dispersed in deionized water (20 mL) was ultrasonically treated to obtain a transparent solution. Then, 2 mL UCNPs was added, and the mixture was stirred overnight to obtain a homogeneous solution. After that, deionized water (40 mL), ethanol (6 mL) and NaOH (0.3 mL, 2 M) were added to the above solution. The system was then transferred to a water bath and heated to 70 °C under vigorous stirring. Several minutes later, TEOS (0.2 mL) was added slowly into the solution and stirred vigorously for 10 min. The resulting solution was centrifuged and the product was washed with ethanol three times. To remove the CTAB template, the obtained sample was transferred to ethanol (50 mL) containing NH_4_NO_3_ (0.3 g) and refluxed at 60 °C for 2 h. Finally, the resultant nanospheres were dried at 60 °C.

### Synthesis of yolk-structured mesoporous silica coated UCNPs (UCNPs@ySiO_2_)

The as-fabricated UCNPs@mSiO_2_ nanospheres were initially dispersed in 10 mL water. Then, a 10 mL solution of MnSO_4_·H_2_O (8 mg mL^–1^) and disodium maleate (10 mg mL^–1^) was added into the solution of UCNPs@mSiO_2_ dropwise under stirring. The system was hydrothermally treated at 180 °C for 3 h. Finally, the solution was centrifuged and the sample was washed with ethanol and deionized water three times. To endow the UCNPs@ySiO_2_ with amino groups, 0.15 mL of APTES was added to 20 mL of PBS with the UCNPs@ySiO_2_ dispersed in and then heated to 45 °C for 8 h under stirring. Finally the sample was collected by centrifugation, washed with ethanol and dried at 60 °C.

### Synthesis of the polyoxometalate (POM) clusters

A facile one-pot approach was used to prepare the POM. First, (NH_4_)_6_Mo_7_O_24_·4H_2_O (2 mmol, 2.47 g) was dissolved in ultrapure water (5 mL) under continuous stirring at 25 °C. Then, a solution (2.5 mL) of NaH_2_PO_4_·2H_2_O (1.17 mmol, 0.18 g) was rapidly added into the reaction system. To obtain the POM clusters at various reduction states, 1 mL of l-ascorbic acid at concentrations of 500, 1000, and 1500 mg mL^–1^ was added dropwise into the system under stirring. The samples at different reduction states were correspondingly labeled as POM-R1, POM-R2, and POM-R3 in comparison to POM-R0, which is the oxidation state with no addition of l-ascorbic acid. After further stirring at 25 °C for 15 min, the resultant clusters were precipitated with 40 mL ethanol, collected by centrifugation, washed with water and ethanol three times, and finally dried in a lyophilizer to obtain cluster powders.

### Modifying UCNPs@ySiO_2_ with POM clusters

PBS (20 mL) with UCNPs@ySiO_2_ (24 mg) and POM clusters (3 mg) dispersed in was magnetically stirred at room temperature for 12 h. After that, POM modified UCNPs@ySiO_2_ were collected by centrifugation. To remove the free POM clusters, deionized water was used to wash the product three times. The obtained product was noted as UCNPs@ySiO_2_–POM. The POM used in this step is at the R3 reduction state.

### Synthesis of PEGylated UCNPs@ySiO_2_–POM

Ethanol (50 mL) with PEG (50 mg) and UCNPs@ySiO_2_–POM (20 mg) dissolved in was magnetically stirred at 60 °C for 24 h. After that, PEGylated UCNPs@ySiO_2_–POM were collected by centrifugation. To remove the unreacted PEG, ethanol and deionized water were used to wash the product three times. The as-prepared product was denoted as PEG/UCNPs@ySiO_2_–POM upconversion nanocapsules (UCNCs) for subsequent biological experiments. For comparison, PEGylated UCNPs@ySiO_2_ were synthesized using a similar method with UCNPs@ySiO_2_ (20 mg) and PEG (50 mg) stirred in 50 mL ethanol at 60 °C for 24 h.

### DOX loading and releasing test

UCNCs (20 mg) were dispersed into a PBS solution of DOX (20 mL, 0.5 mg mL^–1^) under magnetic stirring in a dark room. The same method was used to prepare DOX-loaded UCNPs@ySiO_2_ (UCNPs@ySiO_2_–DOX). The DOX-loaded UCNCs (UCNCs–DOX) were collected by centrifugation after 24 h stirring, and the supernatant was collected to evaluate the DOX loading rate. The precipitated mixture was kept for the further DOX release process. 10 mL of PBS was replenished and set in a water bath kettle at 37 °C with magnetic stirring, and then the supernatant was kept for further UV-vis analysis. At varied time intervals, this release process was repeated in PBS solutions under varied conditions (pH 7.4 and GSH 0 mM, and pH 5.5 and GSH 8.0 mM). To study the NIR influence on DOX release, a PBS solution (pH 5.5, and GSH 8.0 mM) with UCNCs–DOX dispersed in was irradiated with 808 nm light during a release time of 1 to 5 h. The DOX loading rate and the DOX content in the solutions were measured using a UV-vis instrument at a wavelength of 480 nm. The same method was used to investigate the DOX releasing property of the DOX-loaded UCNPs@ySiO_2_–DOX.

### Characterization

A sample for X-ray diffraction (XRD) analysis was prepared by depositing the sample solution on glass slides and vacuum drying at 80 °C. The XRD patterns of the samples were recorded on a D8 Focus diffractometer (Bruker) using CuKa radiation (*λ* = 0.15405 nm). Transmission electron microscopy (TEM) micrographs were obtained using a FEI Tecnai G^2^ S-twin transmission electron microscope with a field emission gun operating at 200 kV. Fourier-transform infrared (FT-IR) spectra were obtained on a Vertex Perkin-Elmer 580BIR spectrophotometer (Bruker), using the KBr pellet technique. The N_2_ adsorption–desorption isotherm and pore-size distribution were tested on a Micromeritics Tristar 3000 instrument. UV-vis absorption spectra were acquired using a TU-1901 dual beam UV-vis spectrophotometer. Upconversion emission spectra were measured on an Edinburgh FLS 980 apparatus, from 400 to 700 nm, using an 808 nm laser diode module as an irradiation source.

### *In vitro* degradation experiment

*In vitro* degradation profiles of the UCNCs were assessed by measuring the accumulated degradation of Mn and Si elements using ICP-OES. Typically, the UCNCs were added into PBS solution (pH 7.4 and GSH 0 mM, and pH 5.5 GSH 8.0 mM). To investigate the NIR influence on biodegradation, PBS solution (pH 5.5, and GSH 8.0 mM) was irradiated with 808 nm light during the biodegradation time of 4 to 8 h. It is noted that all of the evaluation is based on the concentration of 0.1 mg mL^–1^ UCNCs. The testing solution was put into a water bath at 37 °C under slow magnetic stirring. At given time intervals, a small amount of the degradation solution was taken out for ICP-OES testing.

### *In vitro T*_1_-weighted MR imaging

The *in vitro* MR imaging experiments were conducted in a 0.5 T MRI magnet. The UCNCs were diluted into various concentrations. *T*_1_ was acquired using an inversion recovery sequence. *T*_1_ measurements were conducted using a nonlinear fit to changes in the mean signal intensity within each well as a function of repetition time (TR) using a Huantong 1.5 T MR scanner. Finally, the *r*_1_ relaxivity values were determined through the curve fitting of 1/*T*_1_ relaxation time (s^–1^) *versus* the sample concentration (mg mL^–1^). A similar method was used to measure the *T*_1_-weighted MR imaging performance of UCNPs@mSiO_2_.

### *In vitro* and *in vivo* X-ray CT imaging

The *in vitro* CT imaging experiments were performed on a Philips 64-slice CT scanner at a voltage of 120 kV. The sample was diluted into various concentrations and then placed in a line for CT imaging testing. The female mice were first anesthetized with 10% chloral hydrate (0.03 mL g^–1^ of mouse) by intra-peritoneal injection to perform *in vivo* CT imaging. Then, 100 μL of UCNCs saline solution (1 mg mL^–1^) was injected intratumorally into the tumor-bearing mice for scanning. A similar method was used to measure the CT imaging performance of UCNPs@mSiO_2_.

### *In vitro* cellular uptake and UCL microscopy (UCLM) observations

To investigate the cellular uptake process in HeLa cells using a confocal laser scanning microscope (CLSM), the HeLa cells were seeded in a 6-well culture plate and cultured overnight to obtain monolayer cells. After that, 1 mL of UCNCs–DOX (1 mg mL^–1^) was added to the wells and incubated for 0.5, 1, and 3 h, respectively. Then, the cells were washed with PBS three times and stained using DAPI for 10 min. 1 mL of glutaraldehyde (2.5%) was used to fix the cells for 10 min, which were then further washed with PBS three times. Lastly, fluorescence images of the cells were recorded using a Leica TCS SP8 instrument. For the UCLM observation, slides were prepared using the same process except that the images were recorded using an inverted fluorescence microscope (Nikon Ti–S), and an external continuous wave 980 nm laser was used to radiate the samples.

### *In vitro* cytotoxicity

A typical MTT assay was used to evaluate the *in vitro* cytotoxicity. To assess the cytotoxicity of the UCNCs–DOX against cancer cells, HeLa cells (6000–7000 well^–1^) were seeded in a 96-well plate and cultured in a humid incubator (37 °C, and 5% CO_2_) for 24 h. POM clusters and UCNCs–DOX were dispersed into the culture media at concentrations of 0, 15.6, 31.2, 62.5, 125, 250, and 500 μg mL^–1^, and then the cells were treated with NIR irradiation, POM + NIR, UCNCs–DOX, and UCNCs–DOX + NIR, respectively. The materials were added and incubated for 6 h to complete the cell uptake, and then irradiation was carried out. After that, 20 μL of MTT solution (5 mg mL^–1^) was added into each well. After incubation for 4 h, 150 μL of DMSO was added to the wells and the absorbance at 490 nm was recorded for calculation. The percentage of viable cells in the experimental group to that in the control group was used to express the cytotoxicity. The *in vitro* viability of the POM clusters and the final UCNCs to L929 fibroblast cells was also assessed using a similar MTT assay. The concentrations of the samples were 15.6, 31.2, 62.5, 125, 250, and 500 μg mL^–1^. The same method was used to investigate the *in vitro* cytotoxicity of DOX-loaded UCNPs@ySiO_2_ without and with NIR irradiation.

Also, HeLa cells were seeded in a 6-well culture plate and grown overnight. Subsequently, four wells of cells were treated with NIR irradiation, POM + NIR, UCNCs–DOX, and UCNCs–DOX + NIR, respectively. The samples were added and incubated for 6 h to complete the cell uptake, and then the irradiation was carried out. After the treatment, the wells were washed with PBS dyed with both calcein AM and PI, and visualized using CLSM. The pump power of the NIR irradiation was 0.72 W cm^–2^.

### *In vivo* toxicity

A tumor xenograft was planted in the left axilla of each female mouse (15–20 g). The mice were purchased from the Second Affiliated Hospital, Harbin Medical University, and all of the mouse experiments were performed in compliance with the criteria of The National Regulation of China for Care and Use of Laboratory Animals, and approved by the Harbin Science and Technology Bureau. When the tumor size was about 6–8 mm, the mice were randomly divided into four groups (*n* = 5 group^–1^). The first group was used as a control group with saline injection. The residual three groups were treated with POM + NIR, UCNCs–DOX, and UCNCs–DOX + NIR, respectively. For the NIR irradiation process, the tumor site was irradiated with an 808 nm laser for 10 min, 4 h after injecting the sample. The body weights and tumor sizes were recorded every 2 days after the treatment. Tumor growth was recorded by measuring the perpendicular diameter of the tumor with calipers. Tumor volume (mm^3^) was calculated as *V* = *lw*^2^/2, in which *l* and *w* represent the length and width of the tumor.

### Histological examination

After 14 days of therapy, the histological analysis was carried out. Less than 1 cm × 1 cm of tissue of the liver, lung, kidney, heart, spleen and tumor of the representative mice in four groups were excised. Then, the excised tissues were successively dehydrated using buffered formalin, ethanol at varied concentrations, and xylene. Finally, the above dehydrated tissues were embedded using liquid paraffin, and sliced for hematoxylin and eosin (H&E) staining. The stained slices were examined using an optical microscope.

## Conflicts of interest

There are no conflicts to declare.

## Supplementary Material

Supplementary information
