# Supplementary material for: Bioresponsive and near infrared photon co-enhanced cancer theranostic based on upconversion nanocapsules
Source: Chem Sci. 2018 Feb 6;9(12):3233–47. doi: 10.1039/c7sc05414a (PMC5931193; doi:10.1039/c7sc05414a)
Supplement: Supplementary file 1 [file SC-009-C7SC05414A-s001.pdf]

Electronic Supplementary Material (ESI) for Chemical Science.
This journal is © The Royal Society of Chemistry 2018

Bioresponsiveness and near infrared photon co-enhanced cancer theranostic based on upconversion nanocapsules

Jiating Xu,[a] Wei Han,[a] Ziyong Cheng,[b] Piaoping Yang,*[a] Huiting Bi,[a] Dan Yang,[a] Na Niu,[c] Fei He,[a] Shili Gai,[a] and Jun Lin*[b]

[a] Key Laboratory of Superlight Materials and Surface Technology, Ministry of Education, College of Material Sciences and Chemical Engineering, Harbin Engineering University, Harbin, 150001, P. R. China

[b] State Key Laboratory of Rare Earth Resource Utilization, Changchun Institute of Applied Chemistry, Chinese Academy of Sciences, Changchun 130021, P. R. China

[c] College of Sciences, Northeast Forestry University, Harbin 150050, P. R. China

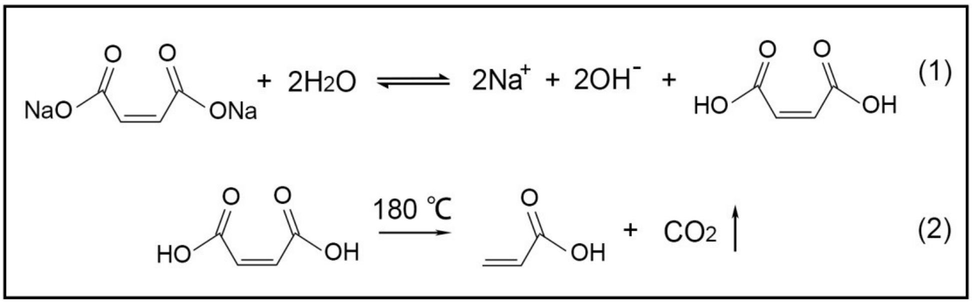

Scheme S1. The reactions between disodium maleate and water resulting in the formation of UCNCs: (1) causing gentle alkalescent solution environment and further promoting hydrolysis of silica shell and (2) generating CO_2 as gas-liquid templates.

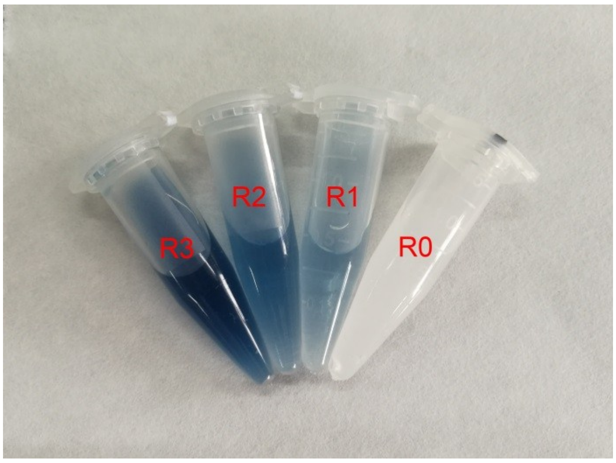

Fig. S1 Photographs of POM cluster at various reduction states dispersed in deionized water.

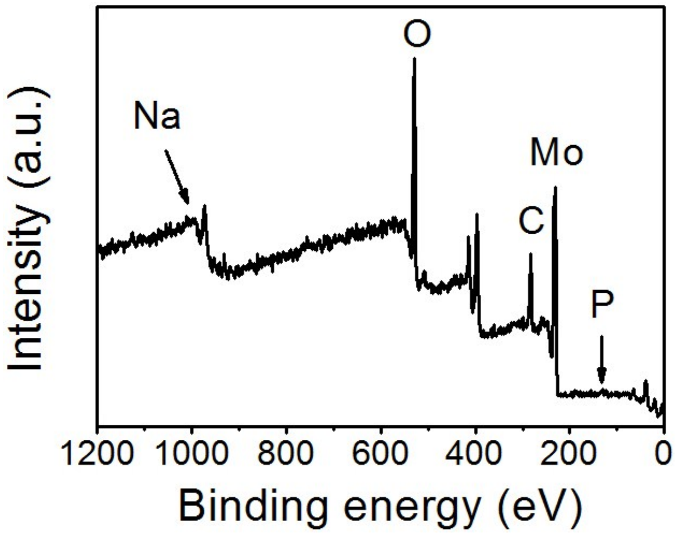

Fig. S2 XPS spectrum of POM-R3 cluster.

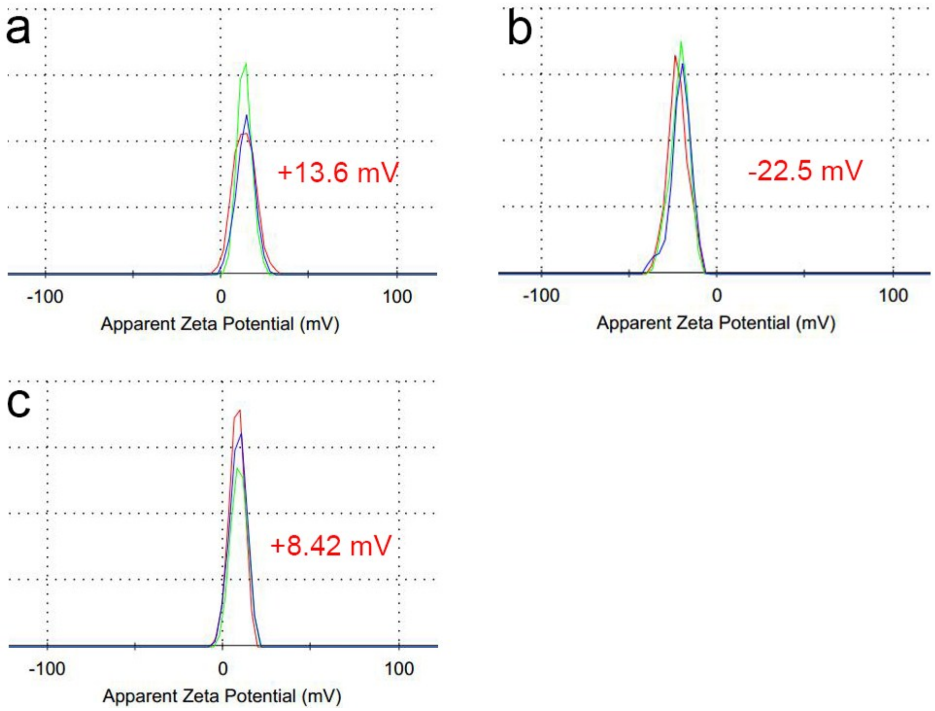

Fig. S3 Zeta potential tests of the amino-modified UCNPs@ySiO_2 (a), POM-R3 cluster (b), and UCNPs@ySiO_2-POM (c).

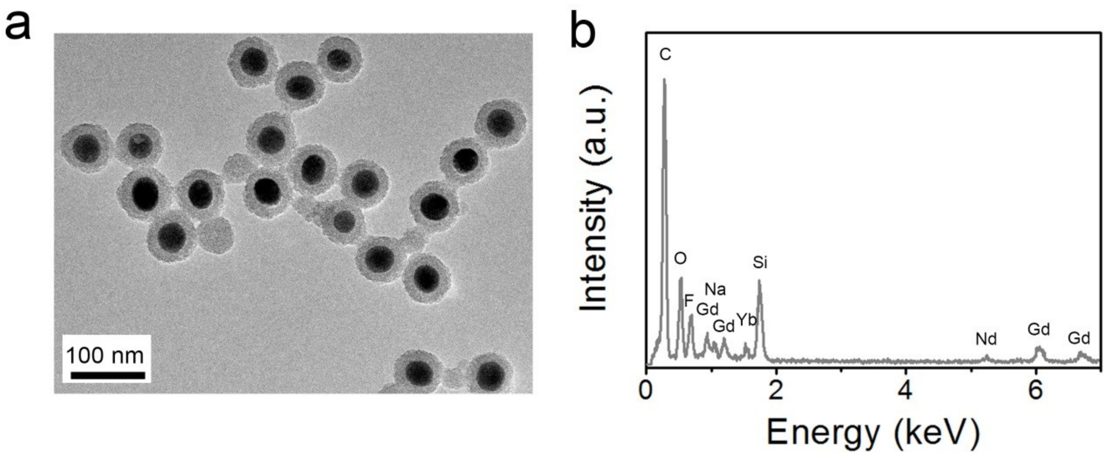

Fig. S4 TEM image (a) and EDS spectrum (b) of UCNPs@mSiO_2 nanospheres.

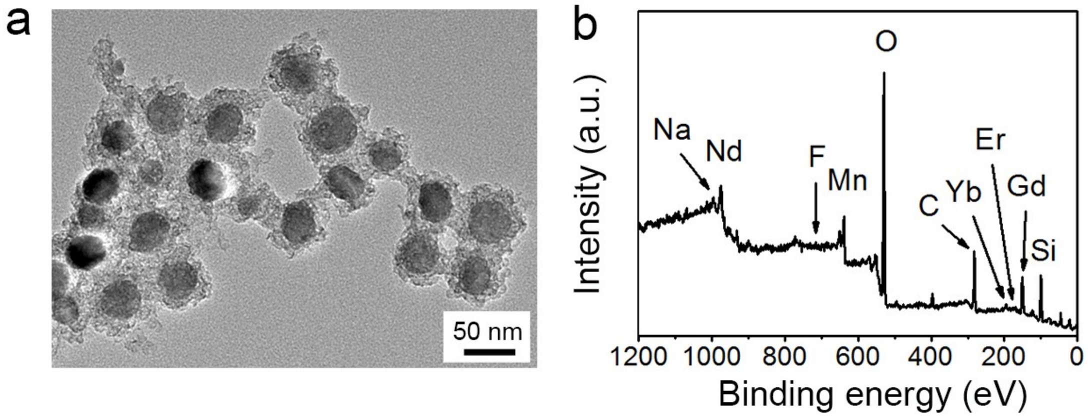

Fig. S5 TEM image (a) and XPS spectrum of UCNPs@ySiO$_2$ (b).

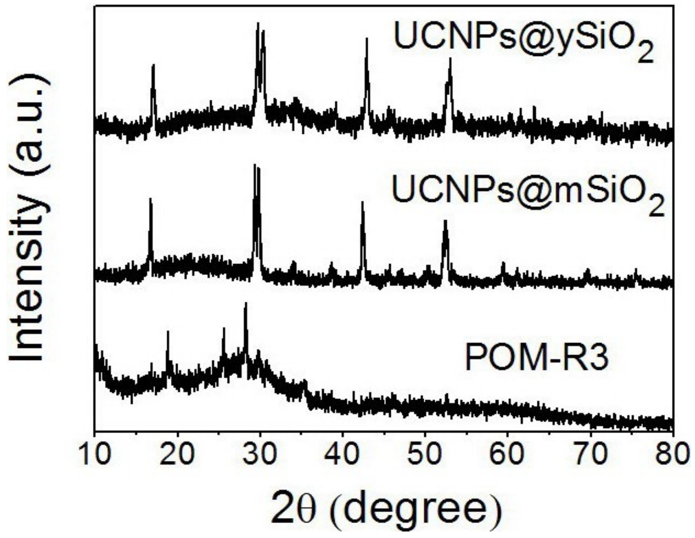

Fig. S6 XRD patterns of the POM-R3 cluster, UCNPs@mSiO_2 and UCNPs@ySiO_2.

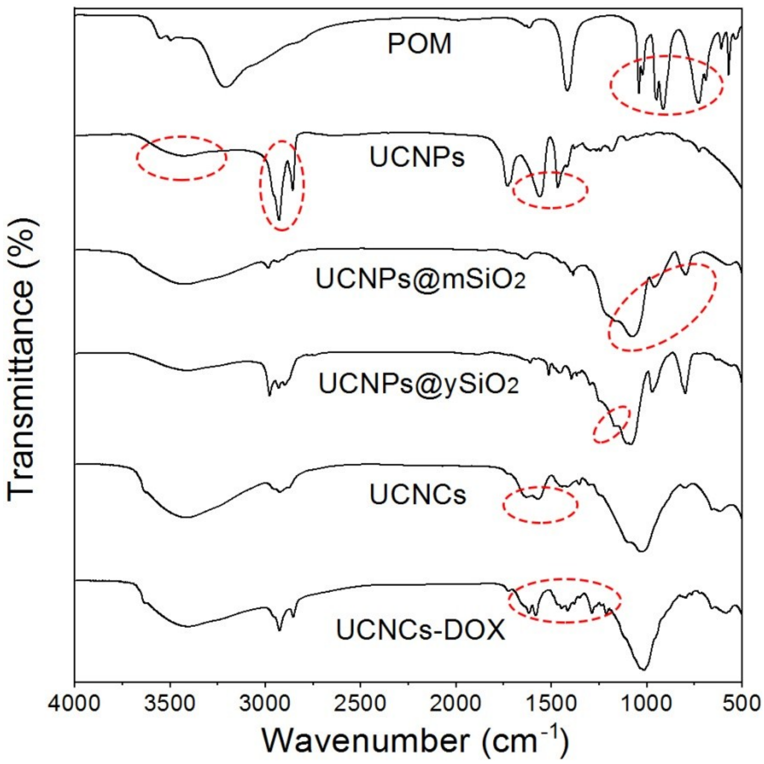

Fig. S7 FT-IR spectra of the prepared POM, UCNPs, UCNPs@mSiO_2, UCNPs@ySiO_2, UCNCs and the final UCNCs-DOX.

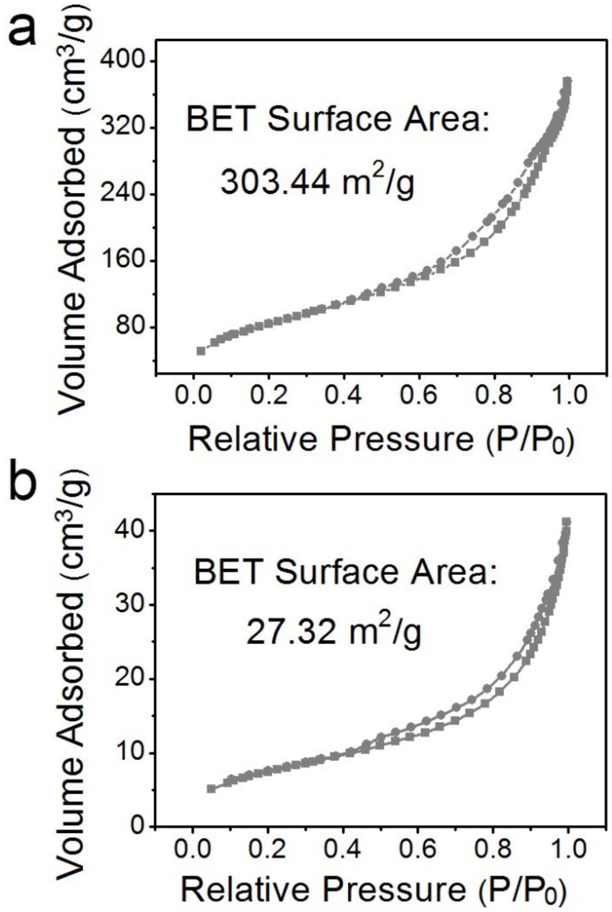

Fig. S8 N_2 absorption-desorption isotherm of the UCNPs@ySiO_2 (a) and the UCNCs-DOX (b).

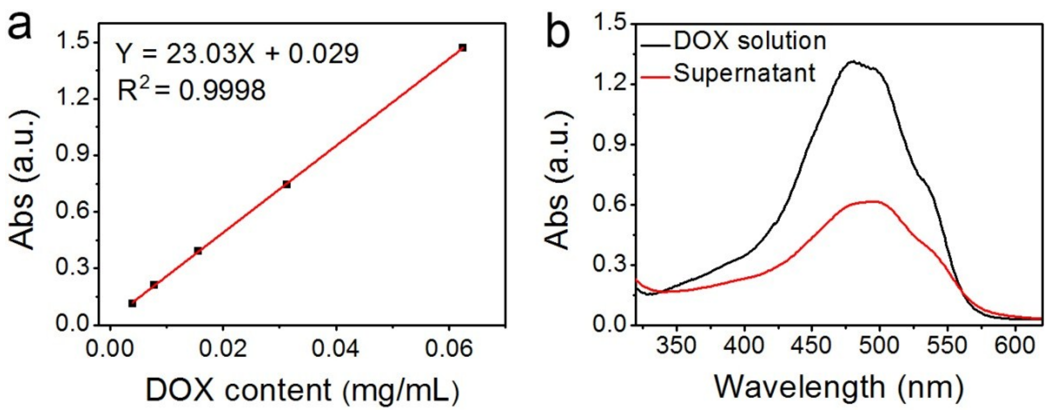

Fig. S9 The standard curve for DOX solution detected at 480 nm (a) and the absorbance spectra of the initial DOX solution and the supernatant obtained after drug loading process with UCNCs (b).

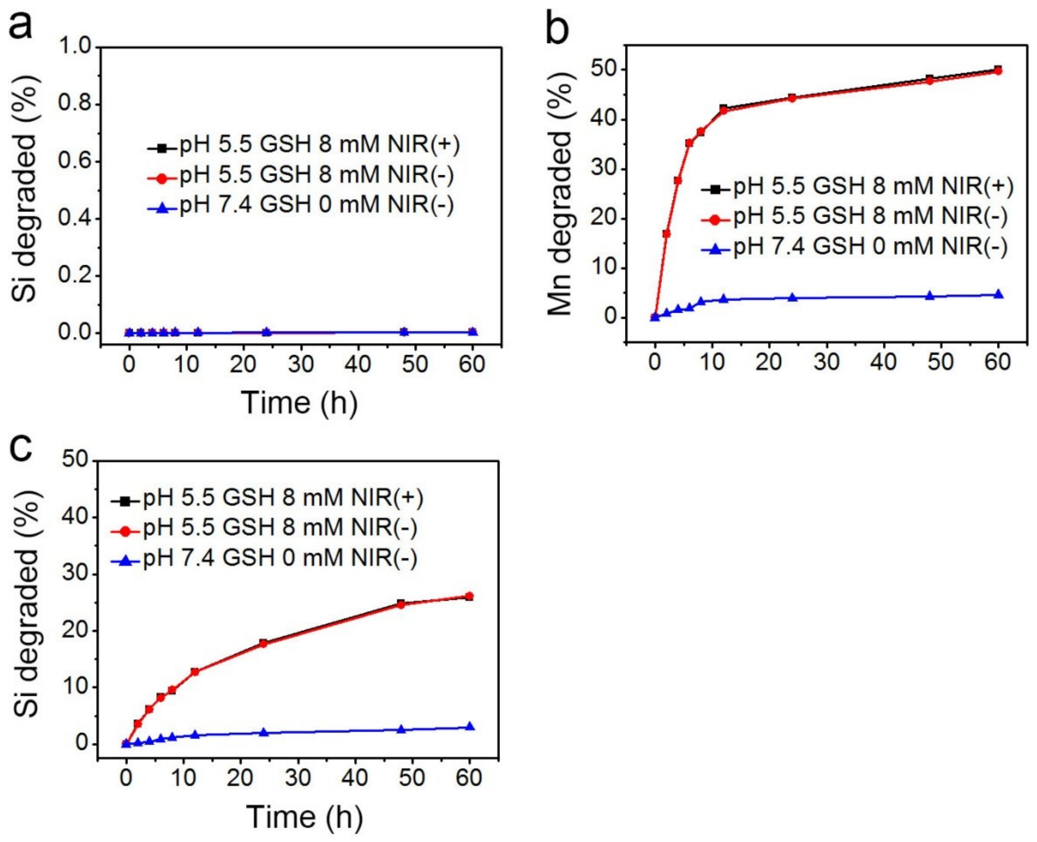

Fig. S10 Accumulated release profiles of biodegraded Si from UCNPs@SiO_2 (a), biodegraded Mn (b) and Si (c) from UCNPs@ySiO_2 in PBS (pH 7.4, GSH 0 mM and pH 5.5, GSH 8 mM) without and with NIR laser irradiation from 4 to 8 h.

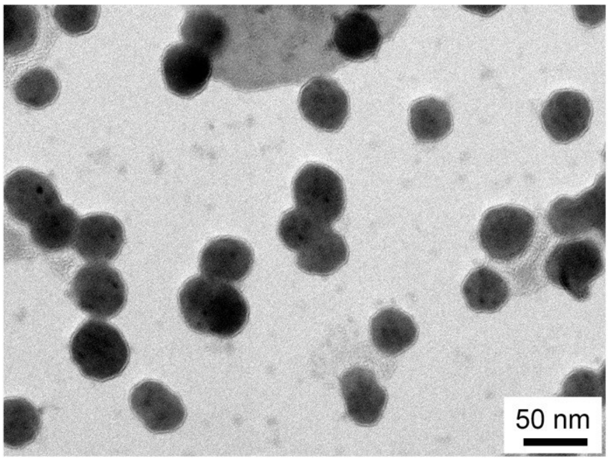

Fig. S11 TEM image of UCNCs after biodegradation in PBS (pH 5.5, GSH 8 mM) for 24 h (the solution was irradiated by 808 nm laser from 4 to 8 h).

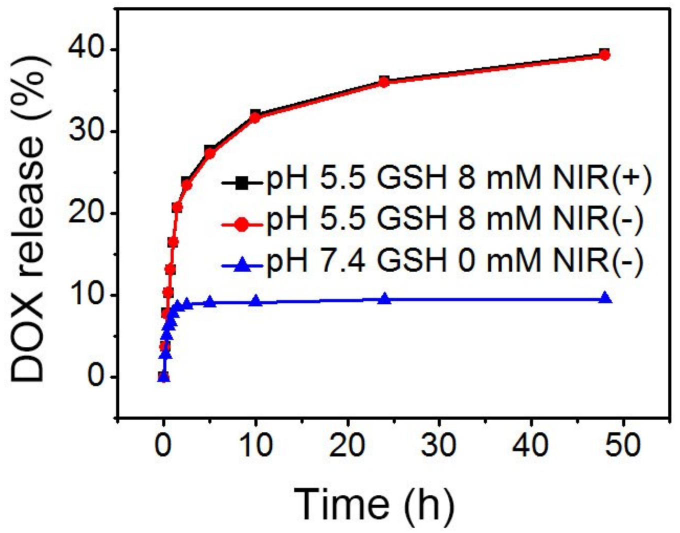

Fig. S12 DOX release profiles from UCNPs@ySiO$_2$-DOX in PBS (pH 7.4, GSH 0 mM and pH 5.5, GSH 8 mM) without and with NIR laser irradiation from 1 to 5 h.

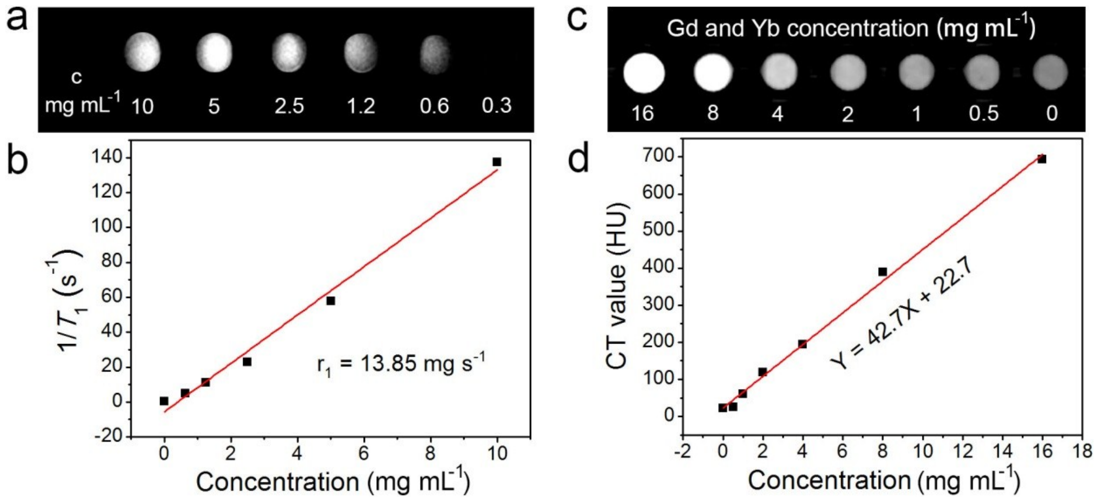

Fig. S13 *In vitro* T_1-weighted MR images of UCNPs@mSiO$_2$ (a) and corresponding relaxation rate R_1 *versus* sample concentrations (b). *In vitro* CT images of UCNPs@mSiO$_2$ at different Gd/Yb concentrations (c) and corresponding CT values *versus* Gd/Yb concentrations (d).

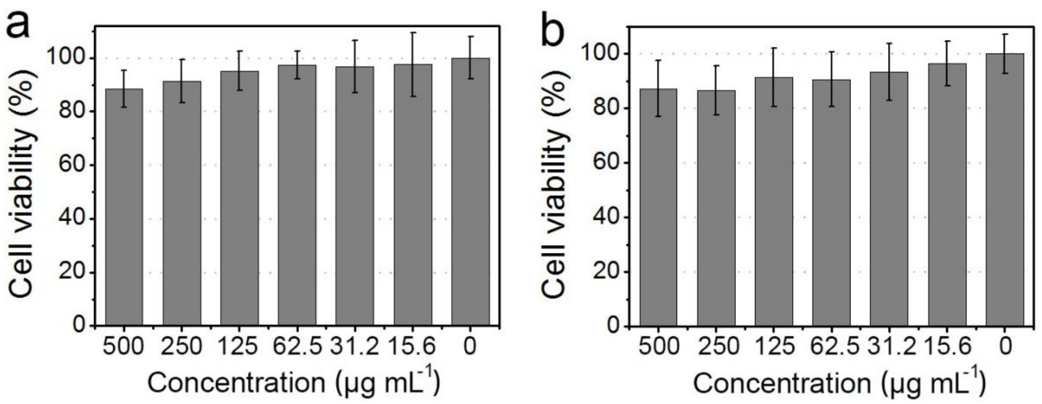

Fig. S14 L929 fibroblast cell viability after incubating with POM (a) and the final obtained UCNCs (b) for 24 h and quantitative assays by standard MTT method.

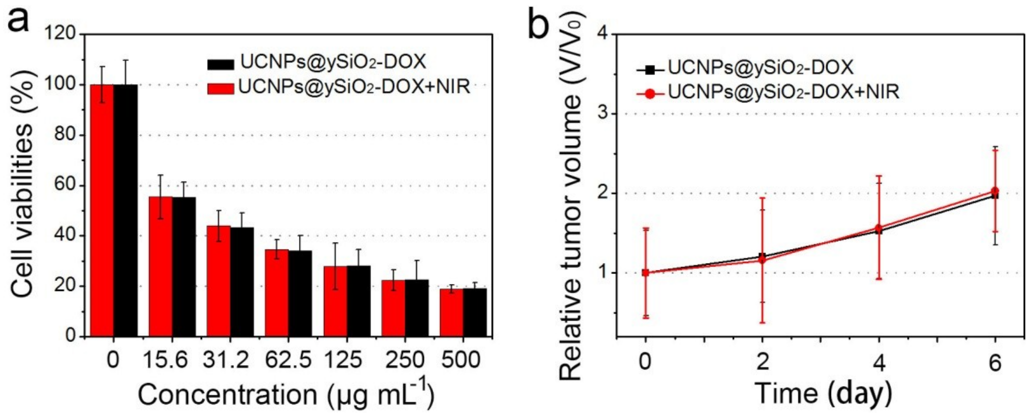

Fig. S15 Viabilities of HeLa cells after incubation with UCNPs@ySiO_2-DOX without and with NIR irradiation (a). Changes in the relative tumor volume achieved from the mice after treatment of UCNPs@ySiO_2-DOX without and with NIR irradiation in 6 days.

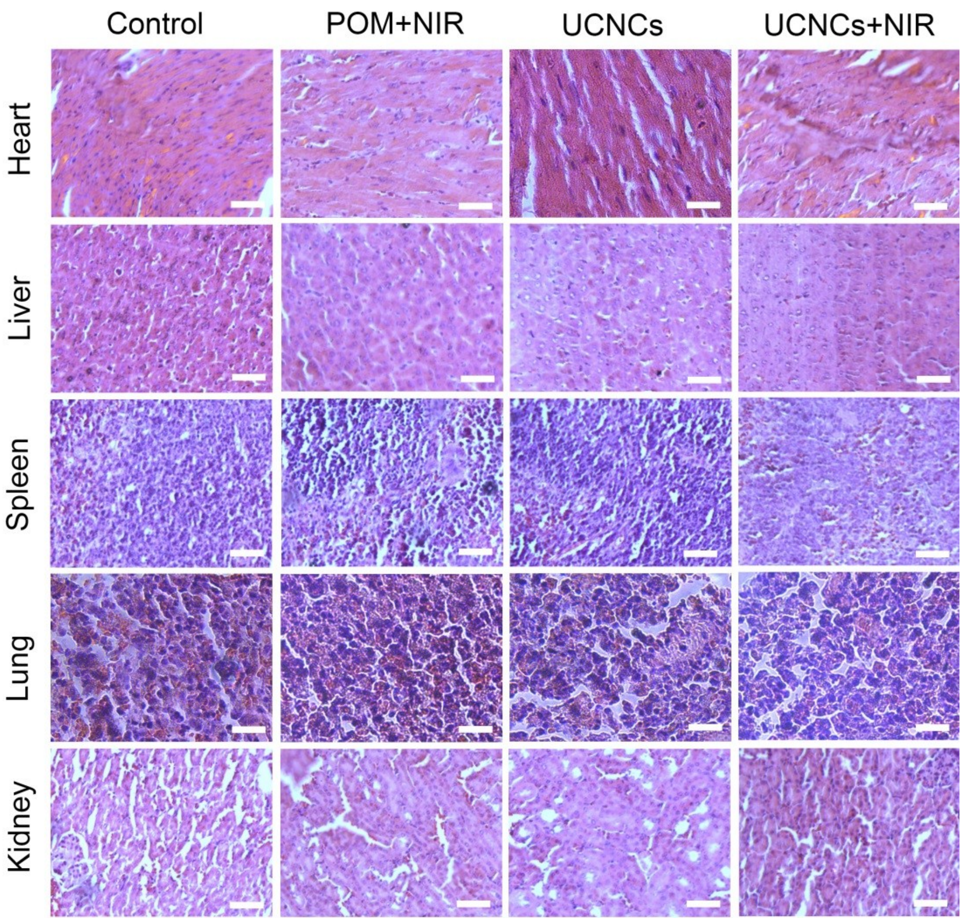

Fig. S16 H&E stained images of liver, lung, kidney, heart and spleen achieved from different groups after 14 days treatment. Scale bar: 30 μm.

Table S1. Blood biochemistry and hematology data of female mice

Project Name	Treatment Group<br>Mean ± SD	Control Group<br>Mean ± SD	units
ALT	42.08±5.12	43.11±4.12	U/L
AST	154.21±17.32	151.23±16.23	U/L
ALP	133.1±14.1	141.2±18.1	U/L
A/G	0.4 ± 0.02	0.4 ±0.01	
BUN	6.17±0.58	6.23±0.67	mmol/L
WBC	12.14±1.48	12.32±1.65	10^9/L
RBC	9.87±0.42	10.14±0.48	10^{12}/L
HGB	164.31±1.65	165.13±2.15	g/L
PLT	839.08±41.12	838.65±43.25	10^9/L
HCT	47.1 ± 1.6	46.4 ± 2.3	%
MCV	54.21±4.14	53.51±3.08	fL
MCH	16.42±0.79	16.17±0.47	pg
MCHC	314.65±2.85	315.68±3.18	g/L

Notice: the data in the table is average calculated by five mice each group. Healthy female mice *i.v.* injected with UCNCs were sacrificed at 14 days for blood collection. Serum biochemistry data including blood urea nitrogen (BUN) levels, albumin/globin ratios, and liver function markers: aspartate aminotransferase (AST), alkaline phosphatase (ALP), alanine aminotransferase (ALT), blood urea level (BUN), the ratio of albumin and globulin (A/G), red blood cells (RBC), white blood cells (WBC), mean corpuscular volume (MCV), hemoglobin (HGB), mean corpuscular haemoglobin (MCH), mean corpuscular hemoglobin concentration (MCHC), platelets (PLT), and hematocrit (HCT).
